# What demographic attributes do our digital footprints reveal? A systematic review

**DOI:** 10.1371/journal.pone.0207112

**Published:** 2018-11-28

**Authors:** Joanne Hinds, Adam N. Joinson

**Affiliations:** School of Management, University of Bath, Bath, United Kingdom; ETH Zurich, SWITZERLAND

## Abstract

To what extent does our online activity reveal who we are? Recent research has demonstrated that the digital traces left by individuals as they browse and interact with others online may reveal who they are and what their interests may be. In the present paper we report a systematic review that synthesises current evidence on predicting demographic attributes from online digital traces. Studies were included if they met the following criteria: (i) they reported findings where at least one demographic attribute was predicted/inferred from at least one form of digital footprint, (ii) the method of prediction was automated, and (iii) the traces were either visible (e.g. tweets) or non-visible (e.g. clickstreams). We identified 327 studies published up until October 2018. Across these articles, 14 demographic attributes were successfully inferred from digital traces; the most studied included gender, age, location, and political orientation. For each of the demographic attributes identified, we provide a database containing the platforms and digital traces examined, sample sizes, accuracy measures and the classification methods applied. Finally, we discuss the main research trends/findings, methodological approaches and recommend directions for future research.

## Introduction

We use the internet and digital devices in many aspects of our lives—to communicate, work, shop, bank, etc. Approximately 50% of the world’s population now use the internet [1] and current estimates predict that around 30 billion devices will be connected to each other by 2020 [2]. With every click or online interaction, digital traces (also known as ‘digital footprints’) are created and captured (usually automatically), providing a detailed record of a person’s online activity. This constant generation of digital data provides opportunities to harvest and analyse ‘big data’ at an unprecedented scale and gain insights to an individual’s demographic attributes, personality, or behaviour. Such information can be incredibly valuable for organisations (e.g. marketers, researchers, governments) hoping to understand digital data and predict future outcomes. Computer and data scientists have used digital data to successfully predict events including: the spread of flu in the US [3], box office revenue for new films [4], election results [5] and reactions or opinions to events such as the Arab Spring [6].

Predicting individuals’ demographic attributes has become a rapidly growing area of research in recent years. However, the innumerable attributes, traces and platforms available, combined with diverse methodological approaches means that research is extremely disparate and published in a variety of journals and conference proceedings. In this article we systematically review existing research to address the questions: (i) what demographic attributes can be predicted from digital traces? (ii) what traces and platforms have been studied? and (iii) how effective are current methodologies and predictions? In synthesising this information, we review current findings and offer recommendations for future research.

### Background

Inferring individuals’ demographic attributes has a long history in fields such as computer forensics and marketing. For instance, computer forensic investigators seek to determine the legitimacy of communications and online activities in order to prevent crimes such as bullying, harassment, or the unauthorised conveyancing of information. Marketers seek to establish who people are in order to target products and services to their desired audiences. In some circumstances, inferring certain attributes such as gender, approximate age and ethnicity may be relatively easy if individuals disclose this information or if they are visible in photographs. Conversely, if such information is absent, or if individuals try to masquerade as someone else, inferring attributes accurately becomes much more difficult.

One way of addressing this challenge is to analyse digital traces that ‘objectively reveal’ a person’s identity. For instance, personality researchers have suggested that individuals leave *behavioural residue* (unconscious traces of actions that may objectively depict their identity, e.g. web browsing histories) when they interact online (e.g. [7,8]). Thus, behavioural residue such as language patterns, smartphone metrics and meta-data (e.g. no. posts, no. followers), provide opportunities to infer demographic attributes with computational techniques (e.g. natural language processing, machine learning) that would be too complex for humans to process. To date, numerous studies have predicted demographic attributes accurately from digital traces including Facebook likes [9–11], smartphone logs [12–15], Flickr tags [16], and language-based features [17–20].

Network analysis is another approach that can be useful for attribute inference. Researchers studying social networks often examine if people who are similar in age, interests, location etc. tend to be closely located in their social networks. Homophily–the notion that *birds of a feather flock together* is incredibly useful within this context, because gathering data from a person’s network may improve the predictive accuracy of individuals for whom we have little, or distorted, data. The downside is that highly sensitive, or private attributes may be identifiable from other people’s data. Indeed, this possibility raises numerous ethical and privacy concerns about what true ‘informed consent’ is, and what can be considered ‘personally identifiable information’ when hidden traits can be discovered using a combination of seemingly innocuous unrelated digital traces. For instance, the data analytics company, Cambridge Analytica recently came under scrutiny in the news for using data collected from approximately 87 million individuals’ Facebook accounts without their explicit consent [21]. The data was supposedly used to create targeted advertisements, which attempted to influence people’s voting preferences in the ‘Vote Leave’ campaign in Britain’s European Referendum, and Donald Trump’s 2016 presidential election [21,22]. If we are going to be able to critique such efforts, and identify what information about a person should be considered ‘protected’, then it is important that we know what the current state-of-the-art is in terms of predicting attributes from digital traces. These joint concerns motivate the present systematic review.

Although demographic inference is almost entirely reported in computer science journals and conferences, there is extensive social psychology research that has explored how demographic attributes (particularly gender and age) relate to certain behaviours, such as language [23], technology use [24–26] and social activities [27]. Unfortunately, the two fields tend to remain distinct, with each adopting different conventions in terms of focus, methods and publishing. Computer scientists typically focus on improving methods and prediction outcomes, whereas psychologists aim to understand people’s behaviour. As such, the majority of research identified by our search was published within computer science outlets. However, we seek to bridge this gap, wherever possible by discussing related psychology research. In the following section we outline our methods and search criteria.

## Method

### Search strategy

We systematically searched for articles published up until October 2018 (i.e. our search had a cut-off date of 30^th^ September 2018) using four strategies. First, we performed searches in the Web of Science, IEEE and ACM online libraries for all relevant articles by searching for keywords and topic-related terms. These included (predict* or identify or detect* or Facebook or Twitter or Instagram or YouTube) and (demographic* or age or gender) and (digital or internet or online or computer-mediated) and (social* or web* or mobile* or sms or big data). Second, we identified all first authors with 3 or more papers and individually searched for further relevant papers written by these authors (identified via Google Scholar, Research Gate and their personal university web pages). Third, we hand searched the references of the papers that met our inclusion criteria and retrieved all further references. We performed this step iteratively on each paper added to the set, until no further papers were retrieved. Fourth, experts in the field were contacted to request information about any other studies that we might not have located. The search generated no studies that were in non-English languages. Our search strategy and statistics are reported in accordance with the PRISMA (Preferred Reporting of Items for Systematic Reviews and Meta-Analysis, www.prisma-statement.org) guidelines. The supporting PRISMA checklist is available as supporting information (see the PRISMA checklist included as S1 Table).

### Inclusion criteria

**Fig 1:**
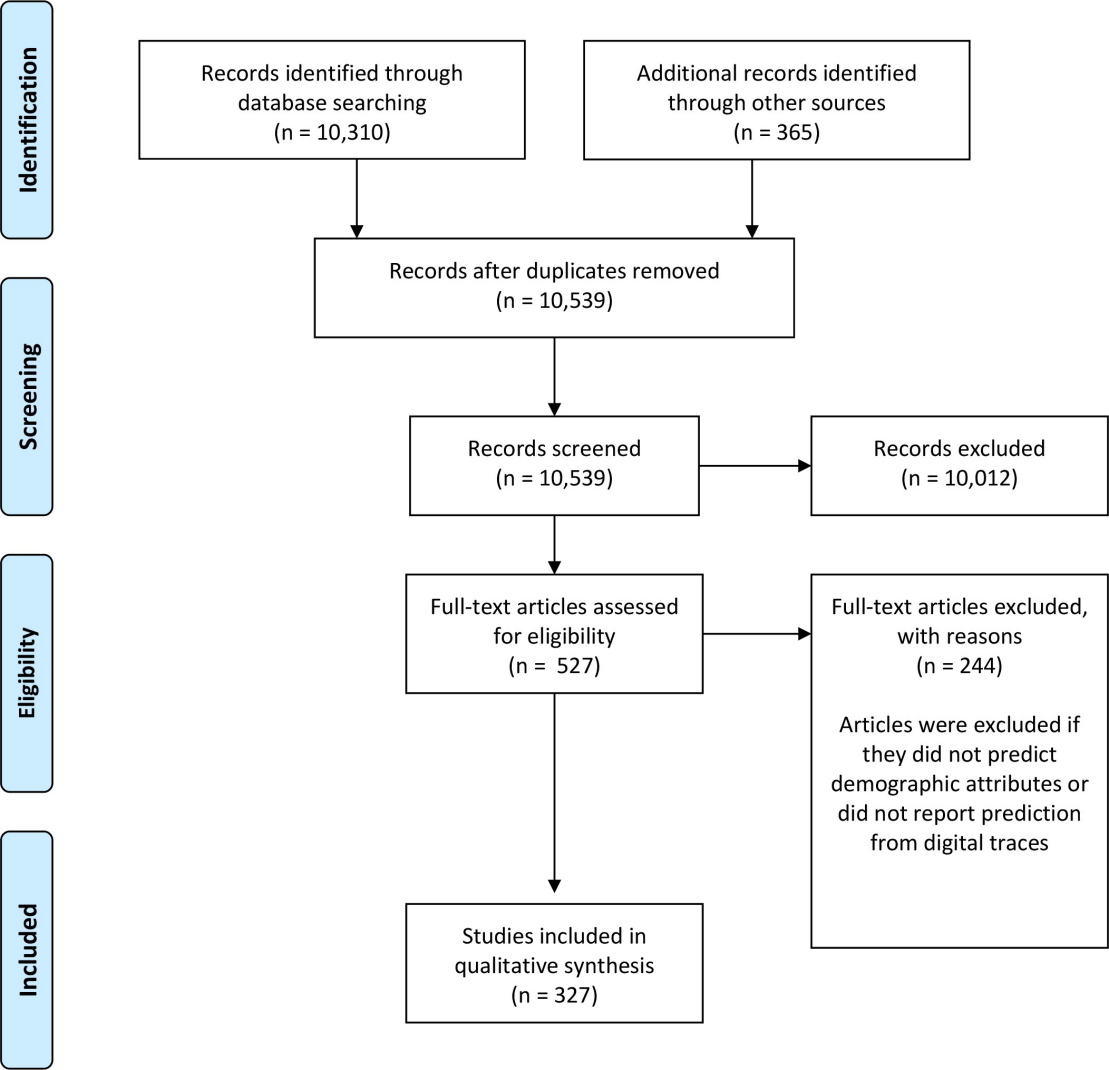
PRISMA Flowchart summarising study retrieval and selection.

To be included in the review, studies had to: (i) report findings where at least one demographic attribute was predicted/inferred from at least one form of digital footprint, (ii) the method of prediction had to be automated—this could include supervised, semi-supervised or unsupervised machine learning and (ii) the digital footprints could either be public (e.g. tweets) or private (e.g. clickstreams). All studies meeting these criteria were included in the review. The search generated a total set of 327 papers. The PRISMA flow chart detailing the papers retrieved and refined according to our criteria is displayed in Fig 1.

### Data collection

For each demographic attribute we extracted the following data from each article: platform and type of digital trace studied, classes used for classification (e.g. unemployed, employed for ‘occupation’; divorced, married, single for ‘family and relationships’), sample sizes, predictive features, accuracy measures (including accuracy (%), area under the ROC curve (AUC), F1-score, precision, and recall), types of classifier used, and publication data (i.e. year of publication, reference data, and the quality of the conference/journal). This data is available as a series of tables in the supplementary materials (S2–S16 for each demographic attribute, respectively).

### Study quality

To our knowledge, there are no existing protocols for assessing the quality of machine learning studies. As such, we assessed the quality of the articles by classifying them on the rank of their publication outlet (i.e. peer-reviewed conference proceedings and journals). We used highly regarded ranking systems of scientific value, specifically the SCImago Journal Rank (SJR) indicator (www.scimagojr.com) for journal articles, and the Excellence in Research in Australia (ERA), Qualis (2012), and Microsoft Academic’s (MSAR 2014) field ratings for conferences databases for conference proceedings. All values were taken from the rankings made in 2018. We scored articles across four categories as follows:

- High quality–journal articles in quartile 1 (Q1), and conference articles ranked as A, A1, or A2
- Medium quality–journal articles in quartile 2 (Q2), and conference articles ranked as B, B1, B2, B3, or B4
- Low quality–journal articles in quartile 3 (Q3) or quartile 4 (Q4), and conference articles ranked as B5, or C.
- Not reported (NR)–journal and conference articles that were not indexed in any of the ranking systems.

We assigned articles that were ranked in multiple categories or quartiles to the highest ranking, for example, articles ranked as B and B5 were classified as ‘medium quality’ (rather than ‘low quality’). A similar approach was used by Azucar, Marengo and Settanni [28] in their review of personality prediction from digital footprints.

## Results

**Fig 2:**
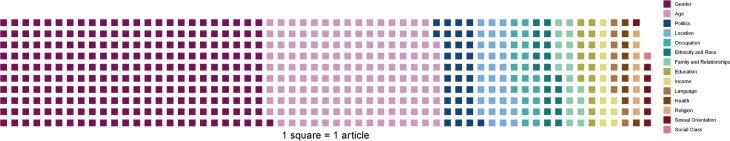
Waffle chart highlighting the proportion of demographic attributes comprising our dataset.

Our search generated a total of 327 articles examining 14 demographic attributes including: gender (n = 241), age (n = 157), location (n = 32), political orientation (n = 33), sexual orientation (n = 7), family and relationships (n = 19), ethnicity and race (n = 20), education (n = 16), income (n = 13), language (n = 9), health (n = 9), religion (n = 8), occupation (n = 22), and social class (n = 1). Many of the articles studied multiple demographic attributes–Fig 2 displays the proportion of attributes studied across our entire dataset.

One of the reasons the number of articles retrieved for gender and age were markedly higher than the other attributes was because of a series of author profiling workshops (PAN) at the Conference and Labs of the Evaluation Forum (CLEF) (https://pan.webis.de). The workshops were held annually and involved teams reporting their solutions to gender and age profiling from a series of provided datasets. The results from the workshops resulted in 105 articles reporting gender, and 63 articles reporting age predictions.

**Fig 3:**
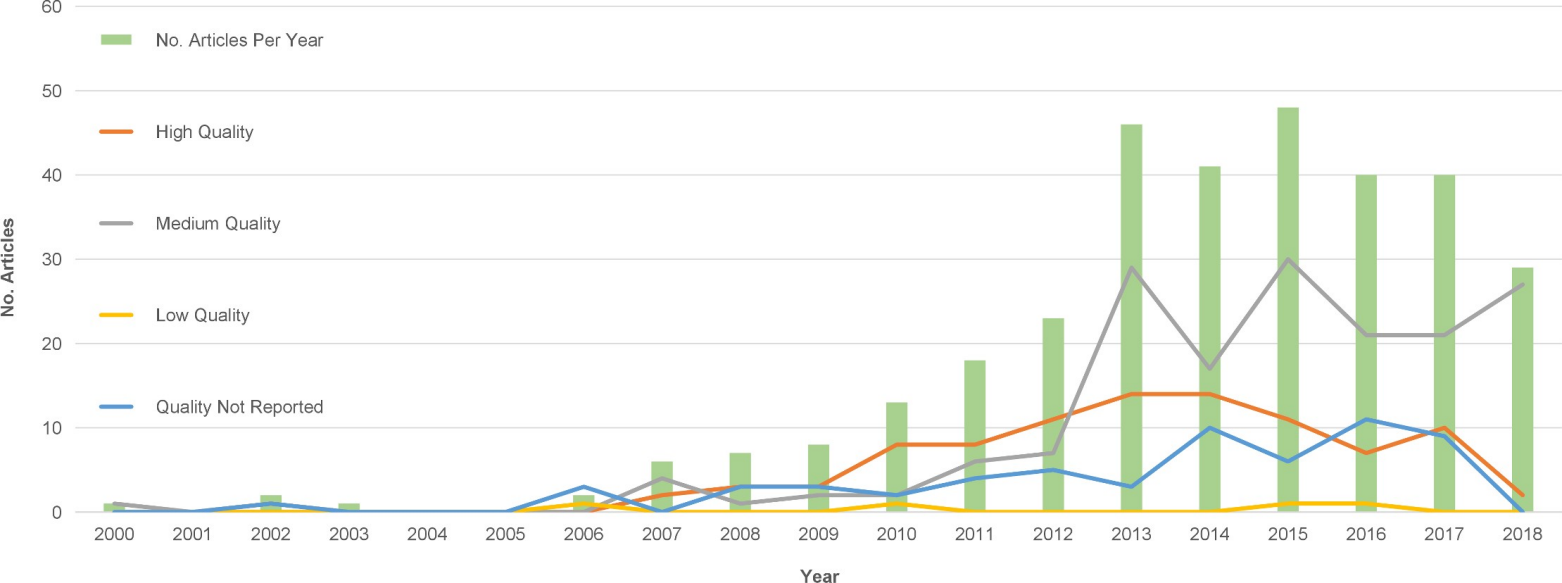
Number of articles published per year and by quality of publication.

Fig 3. displays the number of articles published per year (from 2000 up until Oct 2018) along with number published per quality quartile. The findings highlight that over the last few years, the majority of articles have been published in medium and high-quality journals and conference proceedings. Although a reasonable number of articles were published in journals/conferences that were not indexed in scientific databases, (i.e. we cannot assess the quality of those studies), the number of low-quality articles appears to be very low. In the remainder of this section we discuss the main research findings and trends for each demographic attribute.

### Gender

Gender inference has a long history across numerous disciplines including computer forensics, linguistics and social psychology. In contrast to many other demographic attributes (with the exception of age), extensive research on inferring gender in offline contexts (e.g. conversations, texts, essays) existed prior to the digital-based studies that have proliferated in recent years. As such, it is perhaps unsurprising that gender is the most widely studied attribute within our set (241 articles in total, 136 independent articles, and 105 from the PAN workshops) and is often studied in tandem with age. Table 1 provides an overview of the articles published and associated references per platform. Table 2 provides an overview of the articles published and associated references per predictor. Because of the vast number of articles identified in the search, we discuss the main trends and findings identified over a series of sub-sections, outlined below.

**Table 1:** Number of articles predicting gender, with associated platforms and references.

Category (n = no. articles)	Platform (n = no. articles)	References
**Social Media (134)**	Twitter (106)	[17,20,29–155]
	Facebook (7)	[10,156–161]
	YouTube (2)	[162,163]
	Netlog (2)	[164,165]
	Flickr (3)	[16,166,167]
	Pintrest (1)	[168]
	Instagram (1)	[169]
	Sina Weibo (1)	[170]
	Social Media (General) (25)	[93–100,102,103,127–133,135–142,171]
**Digital Devices (22)**	Smartphones (25)	[12–15,172–192]
	Tablets (1)	[193]
**Websites (23)**	News sites (3)	[194–196]
	Websites (6)	[179,197–201]
	IMDB (1)	[202]
	Hotel Reviews (25)	[93–100,102,103,127–133,135–142,171]
	Movielens (2)	[203,204]
	Crowdfunding Essays (1)	[88]
**Blogs (58)**	Blogger.com (4)	[18,205–207]
	Blogs (General) (51)	[88,93–100,102,103,127–133,135–142,171,208–232]
	Vietnamese Blogs (1)	[233]
	Tumblr (1)	[234]
**Emails (9)**	NR (9)	[196,235–242]
**Radio (3)**	Last.fm (3)	[243–245]
**Search Engines (2)**	Yahoo! (1)	[246]
	Bing (1)	[9]
**Chat (20)**	Chat Logs (General) (18)	[215,216,226–232,217–221,223–225]
	Heaven BBS (2)	[247,248]
**Games (1)**	World of Warcraft (1)	[249]
**Other (18)**	Wi-Fi (1)	[250]
	NA (1)	[251]
	Professional Writing (1)	[88]
	Essays (15)	[127–133,135–142]

**Table 2:** Number of articles predicting gender, with associated predictors and references.

Category (n = no. articles)	Predictors (n = no. articles)	References
**Social Media (134)**	Language (123)	[20,29–65,67,70,72–76,78,80,81,83–89,91–100,102–144,146–149,151–155,158–160,162–165,171,209,214,252–255]
	Network Data (8)	[51,61,62,66,69,78,162,252]
	Colours (4)	[79,90,101,163]
	Meta-data (17)	[61,63,66,69,72,74,78,134,159,171,209,210,252,256–259]
	Names (13)	[29,40,51,69,82,90,112,145,158,161,166,259,260]
	Images (30)	[37–39,41–60,76,77,82,166–169]
	Locations (2)	[29,209]
	Facebook Likes (2)	[10,156]
	Tags (3)	[16,167,169]
	Activity (1)	[169]
	Check-ins (1)	[170]
**Digital Devices (22)**	Application Data (9)	[12–14,172,178,182,188,189]
	Call Logs/SMS Data (11)	[14,15,174,178,181,182,187–189,191,192]
	Location Data (4)	[183–186]
**Websites (23)**	Language (35)	[62,93–100,102,103,127–133,135–142,171,194–196,198,199,202,204,208]
	Website Data (1)	[197]
	Network Traffic Traces (1)	[179]
	Background Colours (1)	[261]
	Video Tags/Titles (1)	[203]
	Web Usage Data (1)	[200]
**Blogs (58)**	Language (55)	[18,93–100,102,103,127–133,135–142,157,171,205–209,211–213,215–221,223–234,262]
	Behavioural Data (1)	[234]
	Meta-data (3)	[66,206,212]
**Emails (9)**	Language (9)	[196,235–242]
**Radio (3)**	Meta-data, Listening Habits (3)	[243–245]
**Search Engines (2)**	Query Log Data (1)	[246]
	Facebook Likes, Profile Data (1)	[9]
**Chat (20)**	Language (20)	[215,216,225–232,247,248,217–224]
**Games (1)**	Behavioural Data (1)	[249]
**Other (17)**	Wi-Fi Traffic (1)	[250]
	Academic Researcher Emails (1)	[251]
	Language (15)	[127–133,135–142]

#### Language

An individual’s choice of language is largely related to their gender, a phenomenon that has been extensively studied by sociolinguists for decades, e.g. [263–265] in written texts, such as essays, poems, scientific articles or speech transcripts, e.g. [266,267]. In general, males and females have been found to differ in numerous ways; typically females tend to use more emotion words, negations and hedges, and males tend to use more assertion, swear words, and long words (over six letters in length), e.g. [268,269]. Lakoff [265] argued that these differences were caused by power differences in society, where women’s lack of power would cause them to adopt more polite and uncertain forms of language. For comprehensive discussions on gender and language, see the work by Coates [270], Lakoff [271], or Holmes and Mayerhoff [272].

On the internet, people’s interactions and communication patterns can change markedly for numerous reasons: a) non-verbal and prosodic cues are lost, b) the design of social media platforms, websites etc. influence the way people converse, and c) individuals may become more conscious of how they present themselves towards others. Digital language traces, combined with computational analytics or tools, such as natural language processing (NLP), and Linguistic Inquiry Word Count (LIWC) [273] enable researchers to study language and gender at mass scale, and in more naturalistic environments. In recent years, gender inference research has grown rapidly, with around 90 of the studies in our set performing some form of predictive analysis across a variety of platforms including Twitter [65,81,84,134], blogs [205,206,211,213], Facebook [157,159,160] and emails [235,236,240]. Researchers have also analysed how language differs by style, [134,205,235,248] sentiment, [74,157,171,255] structure [195,199,235] and content [18,67,84,199].

Overall, research has demonstrated that gender can be predicted from digital traces reasonably successfully, with accuracies often reaching 80% and above [66,72,81,112,193,195,207,209,274]. Studies have highlighted similar trends to offline studies of language, in that females are more likely to use pronouns, emotion words (e.g. happy, bored, love), interjections (e.g. urgh, hmm), while males tend to use more practical dictionary-based words, proper names (e.g. sports team names, numbers and technology words, e.g. [64,160,198,205]. Emoticons (e.g. <3, ☺ and abbreviations (e.g. lol, omg) (which are more often associated with online discourse) tend to be used more frequently by females, whereas males are more likely to post links to websites, videos etc. [67,160]. Gender prediction is also detectable at the level of individual words, word-stems (parts of words) and ngrams (sequences of items or letters, e.g. a unigram = 1 letter, a bigram = 2 letters, a trigram = 3 letters and so forth) e.g. [64,70,72,252,275]. For instance, Mueller and Stuemme [72] found that females tended to use bab, feel and girl (word stems), aa, ah, ee (digrams), and aaa, aha, ee (trigrams), whereas males used scor, team, win (word stems), er, in, re (digrams) and ent, ing, ion (trigrams). S2 Table provides examples of the specific language markers that were particularly successful in predicting gender.

Although these studies have consistently demonstrated trends in gender inference, we should be careful not to generalise the extent to which gender manifests in digital-based language. Most research treats gender as a binary classification task, and attempts to find markers that uniquely identify males and females. However, this disregards evidence and theoretical arguments that gender can be expressed in diverse ways [112], and that gender may manifest differently across social groups, cultures, and contexts. Another consideration is that research is heavily skewed toward inferring gender from English, meaning that there is little exploration of whether these trends extend to other languages. A small number of studies within our set examined other languages including Arabic [194,195], Japanese, Indonesian, Turkish, French [61], Vietnamese [233,276], Russian and Portuguese [63]. The construction of other languages presents numerous challenges–verbs and nouns are either masculine or feminine in French and Spanish for instance, and (to our knowledge), there is less theoretical/social psychology research that explores language-gender differences in other languages and cultures. However, there is evidence to suggest that gender prediction from other languages can be just as successful as English-based approaches. Ciot et al. [61] found that their classifiers which predicted gender from French, Indonesia, Turkish and Japanese tweets achieved similar accuracies to English datasets (with accuracies of 76%, 83%, 87% and 63% for each language respectively). Future research could therefore explore the nuances and effectiveness of gender prediction in other languages.

#### Network data and meta-data

Communications technologies such as social media, smartphones and other digital devices have provoked researchers to question whether an individual’s gender can be predicted from their meta-data (e.g. number of posts, frequency of logins etc.) or through network data derived from their social connections. Researchers often combine such data with language in their classification models in attempt to improve predictive accuracy. In some circumstances, network data have helped to compensate for shortfalls in language-based predictions. For instance, Bamman et al. [260] found that misclassified males and females (i.e. males who were predicted to be female because of their predominant use of ‘feminine’ language and vice versa) were often connected to more members of the opposite gender within their networks. In other words, males who tended to use words commonly associated with females, often had more female followers/friends in their networks and vice versa. As such, males’ different use of language in this context may result from individuals ‘accommodating’ their peers and strong ties by matching their language to maintain and build rapport [277,278].

Other research has used the homophily principle to infer gender directly. For instance, Al Zamal et al. [252] used data extracted from a person’s network neighbours (rather than the individuals themselves) to predict gender on Twitter. Using features such as frequently-used words, stems, ngrams and hashtags, combined with popularity measures of an individual’s network neighbours, Al Zamal et al. [252] inferred gender as accurately as when using the individual’s own data (highest accuracy using network data = 80.02%, accuracy using individual’s own data = 79.50%). Similarly, Jurgens et al. [67] predicted individuals’ gender from their incoming communications (communications directed to an individual), achieving 80% accuracy. Jurgens et al. [67] suggested that because individuals tend to be similar to those in their networks (in terms of their demographic attributes), communication with others often focuses on common ground. This results in reciprocal self-disclosure, meaning that the content, sentiment etc. conveyed by an individual’s friends, also becomes revealing of what an individual may be like.

### Age

The study of age is a vast area of research, encompassing developmental, aging, and social psychology that examines how age is affected by various social processes and how people communicate over their lifespans. Age inference is commonly studied alongside gender and has received much attention from researchers trying to understand how online behaviour may signal how old a person is. Our search generated a set of 157 articles (94 independent articles and 63 articles from the PAN workshops) that reported some form of age inference from digital traces. Table 3 provides an overview of the articles published and associated references per platform. Table 4 provides an overview of the articles published and associated references per predictor. We discuss the main trends and findings for age inference over the following sub-sections.

**Table 3:** Number of articles predicting age, with associated platforms and references.

Category (n = no. articles)	Platform (n = no. articles)	References
**Website (32)**	IMDB (1)	[293]
	Other (8)	[62,171,179,197–199,214,294]
	Hotel Reviews (24)	[93–100,102,103,127–133,135–142]
**Search Engines (2)**	Bing (1)	[9]
	Yahoo! (1)	[246]
**Blogs (54)**	Blogger.com (4)	[18,205,207,295]
	Blogs (General) (50)	[94–100,102,103,127–133,135–142,157,171,209,211,212,214–233,292,296]
	LiveJournal (1)	[297]
**Smartphones (18)**	NR (18)	[13–15,172–174,177–179,181–184,188,189,191,287,298]
**Forums (2)**	Vietnamese Forums (1)	[276]
	Breast Cancer Forum (1)	[292]
**Social Media (84)**	Twitter (75)	[20,51,62,65–67,71,73–75,82,84,87,89,91,93–100,102–111,113–142,171,209,214,252,285,286,290,293,299–303]
	Social Media (General) (25)	[93–100,102,103,127–133,135–142,171]
	Facebook (5)	[10,156,159,160,304]
	Flickr (1)	[166]
	Netlog (2)	[164,165]
	YouTube (2)	[162,163]
	Instagram (2)	[169,289]
	Pokec (1)	[305]
	Sina Weibo (3)	[170,288,306]
**Emails (4)**	NR (4)	[238–241]
**Radio (3)**	Last.fm (3)	[243–245]
**Games (1)**	World of Warcraft (1)	[249]
**Chat (19)**	NR (19)	[215,216,225–232,291,217–224]
**Other (14)**	Essays (14)	[127–133,135–142]

**Table 4:** Number of articles predicting age, with associated predictors and references.

Category (n = no. articles)	Predictors (n = no. articles)	Reference
**Website (32)**	Language (30)	[62,93,103,127–133,135,136,94,137–142,171,198,199,214,95–100,102]
	Website Data (3)	[197,199,294]
	Network Data (1)	[62]
	Network Traffic Data (1)	[179]
	Demographics, Names, Followers (1)	[293]
**Search Engines (2)**	Facebook Likes (1)	[9]
	Query Logs (1)	[246]
**Blogs (54)**	Language (54)	[18,93,103,127–133,135,136,94,137–142,171,205,207,209,95,211,212,214–221,96,222–231,97,232,233,292,295,297,98–100,102]
	Meta-data (6)	[157,207,209,211,212]
**Smartphones (18)**	Application Use (7)	[13,14,177,178,182,188,189]
	Call/SMS Data (15)	[13,14,183,188,189,191,298,15,172,174,177–179,181,182]
	Location Data (8)	[14,173,178,182–184,188,189]
	Accelerometer Data (5)	[14,178,182,188,189]
	Network Data (7)	[15,174,178,179,181,191,287]
**Forums (2)**	Language (2)	[276,292]
**Social Media (84)**	Language (81)	[20,51,84,87,91–98,62,99,100,102,103,105–110,65,111,114–122,66,123–132,67,133–142,71,159,160,162–165,171,209,252,255,73,285,286,288–290,299,300,302,306,307,74,82]
	Meta-data (7)	[66,169,285,289,300,306]
	Network Data (12)	[62,69,216,219,97,98,105,120,154,167,202,211]
	Facebook Likes (2)	[10,156]
	Names (4)	[51,82,166,301]
	Images (4)	[82,166,169,288]
	Check-ins (1)	[170]
**Emails (4)**	Language (4)	[238–241]
**Radio (3)**	Music Meta-data/Listening Habits (3)	[243–245]
	Profile Information (1)	[243]
**Games (1)**	Character Features/Behavioural Data (1)	[249]
**Chat (19)**	Language (19)	[215,216,225–232,291,217–224]
	Meta-data (1)	[291]
**Other (14)**	Language (14)	[127,128,138–142,129–133,135–137]

#### Language

Similar to gender, extensive research has examined how language use is related to age, e.g. [23,279,280] and how a person’s language is influenced by their emotional experiences, identity, social relationships and cognitive abilities over time e.g. [281–284]. Research on age and language has highlighted that individuals’ use of positive emotion, future tense and cognitively complex words (causation words, insight words, long words) tends to increase with age, whereas negative emotion, first-person singular self-references and past tense words tends to decrease [23]. Around 60 articles in our set conducted some form of analysis related to age inferences and language across numerous platforms including Twitter [134,252,255,285,286], websites [197–200], smartphones [13,14,174,178,287], and emails [238,239,241]. Researchers have also analysed how language differs by style [18,74,205,288], content [18,62,67,205,289,290], sociolinguistics [75,255], and ngrams [164,291,292]. S3 Table provides examples of the specific language markers that were particularly successful in predicting age.

Overall, research has demonstrated that age can be predicted from language reasonably successfully, with accuracies often reaching 70% and above [18,164,205,233,252]. Studies have highlighted numerous patterns related to language and age; in terms of content, younger people (in their teens and twenties) used words related to school, work, socialising, computer games and comedians, whereas older adults (over 30) tended to use more family related words and words associated with the news or society [18,62,211]. In terms of style, younger people tended to use more acronyms, slang, self-references, and varied forms of grammar, whereas older adults tended to use more mature and polite language, with less linguistic variation [67,207].

Although these findings seem to broadly align with research on language and age in offline contexts, current methods are quite limited. There is a tendency for researchers to treat age as categorical variables such as 13–17, 18–24, 25–35, and then simply using ‘over 35’ or ‘over 40’ when predicting older ages. This approach can severely undermine the accuracy of prediction, especially for adults over the age of 30 –surprisingly, only 15 studies treated age as a continuous variable, e.g. [160,177,189,287,305]. In some circumstances, obtaining a more approximate age may be acceptable, for instance it is highly unlikely that a person’s choice of product will change vastly from the age of 23 to 24. Another factor that may have hindered research thus far is that younger people tend to use the internet more than older people, so it may have been more difficult to obtain decent ground truth/training data. For instance, a survey by the Pew Internet Centre, highlighted that as of 2018, 66% of US adults over 65 use the internet, compared to 98% of 18-29-year olds. These figures have increased from 14% and 70% respectively since 2000 [308]. Future research may therefore want to consider exploring more nuances in language use across specific ages.

#### Network data

Network data has also been a reliable indicator of a person’s age, with studies highlighting that people of similar ages tend to congregate in the same networks e.g. [177,252,287] and communicate more with each other on social media e.g. [67,209]. Research has also identified patterns of homophily in smartphone records and applications [181,191,287] that varies across different age groups. For example, Park et al. [287] found that children (9 year olds), and teenagers (14-18-year olds) sent most of their SMS messages to others their own age and Dong et al. [191] found that 18–35 year-olds had more (same and opposite gender) contacts than people over 35, who had smaller, same-gender social circles. Similarly, children and teenagers were also identifiable from their communication patterns to people their parents age [287], which subsequently decreased as individuals became older. Although specific explanations from social psychology for these patterns of behaviour do not exist (to our knowledge), these types of findings highlight the potential to gain new understanding and extend existing explanations of how relationships and communication change over different age groups.

### Location

Location-based services (LBS) are incredibly useful across many domains, including personalised services (e.g. local restaurants, hospitals, events), coordinating responses to disease or disasters, and detecting security intrusion. Using digital traces to infer location data enables researchers to examine the relationship between online behaviour and individuals’ locations (e.g. regional nuances, countries etc.), rather than relying upon IP addresses. Because location or geo-location-based work is an area of research within itself, we were careful to restrict our inclusion criteria to studies that predicted location data relating specifically to individuals’ home cities, countries etc. (as opposed to analyses of where individuals were at particular moments in time, e.g. [309]. For articles that cover geolocation prediction in more detail see the work by Jurgens et al. [310] and Stefanidis et al. [311]. 32 articles reported some form of location prediction, across a range of granularities (e.g. home, city, country), platforms (e.g. Twitter, Facebook, Flickr, Foursquare) and traces (e.g. language, network data, location fields in profiles) (see Table 5 and Table 6 for breakdowns of the platforms, predictors and references).

**Table 5:** Number of articles predicting location, with associated platforms and references.

Category (n = no. articles)	Platform (n = no. articles)	References
**Social Media (24)**	Facebook (2)	[156,312]
	Twitter (20)	[29,67,75,82,87,89,313–326]
	Flickr (3)	[16,321,322]
**Location-based Applications (5)**	Foursquare (3)	[67,317,319,327,328]
	Brightkite (1)	[328]
	Google+ (1)	[319]
	Gowalla (1)	[328]
**Blogs (1)**	NR (1)	[233]
**Emails (3)**	NR (1)	[238,239,241]
**Smartphones (2)**	NR (1)	[179,329]
**Forums (1)**	Webretho, Otofun, Tinhte (1)	[276]
**Search Engines (1)**	Yahoo! (1)	[246]
**Websites (1)**	NR (1)	[179]

**Table 6:** Number of articles predicting location, with associated predictors and references.

Category (n = no. articles)	Predictor (n = no. articles)	Reference
**Social Media (24)**	Location Data (16)	[19,31,322,69,312,314–319]
	Network Data (7)	[67,82,312,315,316,318,323]
	Names (2)	[29,82]
	Facebook Likes (1)	[156]
	Language (16)	[67,75,82,87,89,255,313–318,323–326]
	Spatial, Visual, Temporal Features (1)	[321]
**Location-based Applications (5)**	Check-in Data (2)	[327,328]
	Location Data (3)	[67,317,319]
**Blogs (1)**	Language (1)	[233]
**Emails (3)**	Language (1)	[238,239,241]
**Smartphones (2)**	Applications (1)	[179,329]
**Forums (1)**	Language (1)	[276]
**Search Engines (1)**	Query Logs (1)	[246]
**Websites (1)**	Network Traffic Traces (1)	[179]

Inferring location accurately can be challenging due to the complexity of information available, individuals’ personal circumstances and platform design. These challenges have been acknowledged in much of the research conducted to date. For instance, many applications enable individuals to self-report their location–Facebook provides the “Current City” and “Hometown” fields, and Twitter provides the profile “Location” field. Often these fields are non-compulsory, and have no restrictions; as such, individuals can enter incorrect, non-existent or even fake information. For instance, Hecht et al. [313] found that 34% Twitter users did not provide location information in their profiles, and those that did rarely provided detail beyond their current city. Users who did provide data often replaced locations with false places (e.g. “outta space”), sarcastic comments (e.g. “redneck hell”) or celebrities’ names (e.g. “Justin Bieber’s heart”). Despite the limited reliability of profile location fields, numerous studies have used them in their algorithms, but typically in combination with other digital traces such as network data [312] name data [29] and tweet contents [315,316]

Other approaches have involved inferring location solely from language without considering other geospatial cues ([315,326,330]. Language may reveal aspects of an individual’s demographic location if they directly reference particular venues, places or use certain colloquialisms or slang. For instance, people from Texas may use “howdy” frequently, or people from London may reference Arsenal Football Club. Chang et al. [315] and Cheng et al., [325] predicted individuals’ cities tweet location-related contents; their most accurate predictions were 50.93% (within a 100 mile radius) and 78.80% (within a 536 mile radius) respectively. Chang et al.’s method was particularly useful as it only required 250 local words, (selected by unsupervised methods) in contrast to Cheng et al.’s approach which relied on 3,183 local words (selected by supervised classification based on 11,004 hand-annotated ground truth data).

Although these studies have demonstrated that inference from tweet content alone is possible, the language contained within tweets can be very noisy, as people may discuss varied topics and may use language that does not readily link to specific locations (e.g. conjunctions, prepositions, adjectives, or generic terms like ‘restaurant’, ‘city centre’). Network data may therefore provide a more objective measure for predicting location. Numerous studies incorporated various forms of network data in their models including ‘friends” location data [312,320] or network data combined with tweet contents or other meta-data, e.g. [82,310,315]. Traditionally, one would predict that people would tend to know (or be ‘friends’ with) more people in close physical proximity to themselves, that is, they would be connected to people who live in the same town or city. Although the internet has the ability to change this drastically, by connecting people over vast distances, research has highlighted that homophily still holds within this context. Backstrom et al. [312] for instance found that the likelihood of friendship reduced as a function of distance, and their model based on network associations and address data was able to predict the locations of 69.10% of users within a 25-mile radius.

Finally, while the bulk of research has used Twitter data, other studies have examined other platforms and devices, including smartphone applications [329] web traffic data [244] Foursquare e.g. [310,317,328] and Google+ [319]. Foursquare in particular, is designed to provide users with personalised, location-based recommendations, based on their browsing histories, purchases and check-in behaviour. Findings to date have demonstrated accuracies of 67.41% for city [319,327], 80.92% for state, and 93.67% for country-level prediction [327].

### Political orientation

In recent years, the internet has become a hotbed for publishing and promoting political activity. Social media in particular has become a forum where news stories are circulated, political parties disseminate their agendas, and where any individual can express political opinions and beliefs. As such, research exploring political related activity online has proliferated, with researchers attempting to use online data to understand people’s political sentiments e.g. [331,332] and predict election outcomes, e.g. [333,334]. Thus, inferring an individual’s political orientation from their digital traces is just one area among a rapidly growing field of research. Our search generated 33 articles that inferred political orientation from digital traces. Twitter is the most studied platform, with language and network-based features most commonly used for inference (see Table 7 and Table 8 for overviews).

**Table 7:** Number of articles predicting political orientation, with associated platforms and references.

Category (n = no. articles)	Platform (n = no. articles)	References
**Social Media (25)**	Twitter (25)	[19,20,293,299,302,335–341,62,342–346,75,82,84,86,252,255,274]
	Facebook (2)	[10,11]
**Websites (3)**	IMDB (1)	[293]
**Search Engines (1)**	Bing (1)	[9]
**Blogs (4)**	Digg (1)	[347]
	Blogs (Other) (3)	[348–350]

**Table 8:** Number of articles predicting political orientation, with associated predictors and references.

Category (n = no. articles)	Predictors (n = no. articles)	References
**Social Media (25)**	Meta-data (4)	[252,335,337,342]
	Language (24)	[19,20,299,302,335–342,62,343–346,75,82,84,252,255,274,293]
	Network Data (10)	[62,82,86,252,293,299,336,339,340,344]
	Facebook Likes (2)	[10,11]
**Websites (3)**	Language (3)	[62,293,351]
	Network Data (2)	[62,293]
	Location Data (1)	[293]
	Name Data (1)	[293]
**Search Engines (1)**	Facebook Likes (1)	[9]
**Blogs (4)**	Language (4)	[347–350]

Inferring an individual’s political orientation accurately is particularly challenging because it can vary in strength and change over time. This is particularly pertinent when external factors, such as societal events or political campaigns directly attempt to sway peoples’ ideologies. However, the subjective nature individuals’ political preferences has generally not been reflected in existing research. The majority of studies in our set have treated prediction as a classification problem, where individuals are categorised into two [10,86,252,338], three [336,337], or four classes [341,342]. Given that most countries tend to be dominated by two political parties, these approaches may seem logical for gaining a simplistic overview of individuals’ political preferences. However, the disadvantage is that such categorisations cannot capture the strength or idiosyncrasies of individuals’ beliefs. Barberá [86] directly attempted to address this problem by developing a model that estimated ideology on a continuous scale. By using social ties (i.e. who individuals follow), Barberá [86] successfully inferred ideological alignment (strength in terms of right vs. left leaning) across European countries and the US, that correlated strongly with offline measures of voting records. As such, Barberá’s method has since been widely adopted by other political scientists analysing political behaviour online, e.g. [352,353].

Another challenge for predicting political orientation is that gaining valid ground truth is often difficult. Many individuals do not explicitly state their political affiliation online, and those that do are likely to be more politically opinionated or active that the average person. For instance, Priante et al. (2016) claimed that fewer than 5% of Twitter members state their affiliation. Cohen and Ruths [338] suspected this may have caused studies that used explicit political preferences as ground truth to be biased in favour of political activists or those with strong political views. To examine this, Cohen and Ruths [338] constructed three separate Twitter datasets (comprising tweets and hashtags), each representing different strengths of political orientation: a) US politicians’ accounts, b) users who self-reported their political orientation in their accounts, and c) ‘modest’ users who frequently mentioned politics in their tweets, (such that their orientation could be manually inferred), yet without any explicit declaration.

Cohen and Ruths’ [338] findings demonstrated that classification accuracy decreased as visible political engagement decreased. In other words, US politicians’ preferences were the easiest to predict, with 91% accuracy, followed by politically active users at 84% and modest users at 68%. Given that much of the previous research used self-reported political affiliation as ground truth, e.g. [252,255,340], these findings suggested that many of the reported accuracies were likely unrepresentative of the general population. Cohen and Ruths examined this further by testing the transferability of their classifiers and found that accuracy reduced significantly–to 11% when classifiers trained on political figures were tested on modest users.

Perhaps due to Cohen and Ruth’s (somewhat concerning) findings, subsequent research has adopted more cautious approaches toward classification. Preotiuc-Pietro et al., [19] created a language-based model using individuals’ self-reported orientation, where individuals rated the strength of their political ideologies on a seven-point scale (ranging from ‘Very Conservative’ to ‘Very Liberal’). This enabled them to account for varying strength of political preferences rather than limiting predictions to 2–3 classes. Similarly, obtaining self-reports in this instance enabled them to avoid the biased and unrealistic forms of data inherent in the previously used methods. Their accuracies ranged from 22–27%, highlighting that realistic, fine-grained political orientation is more nuanced and complex than that reported by previous research. Future research may therefore want to be mindful of selecting appropriate training data and examining degrees of political orientation to ensure that predictions are realistic.

### Sexual orientation

To date, research on inferring sexual orientation has received little attention in comparison to other demographic attributes, with 7 studies generated from our search (see Table 9 and Table 10). Despite this, inferring an individual’s sexuality has many important implications, especially with regards to individuals’ privacy and how their data may be used. Across many types of social media, individuals have freedom over whether to disclose their sexual preferences, whereas in other platforms such as dating websites/applications, individuals may be required to provide such data in order to use the service.

**Table 9:** Number of articles predicting sexual orientation, with associated platforms and references.

Category (n = no. articles)	Platform (n = no. articles)	References
**Social Media (6)**	Friendster (2)	[354,356]
	Facebook (3)	[10,11,355]
	Sina Weibo (1)	[170]
**Dating Website (1)**	NR (1)	[357]

**Table 10:** Number of articles predicting sexual orientation, with associated predictors and references.

Category (n = no. articles)	Predictors (n = no. articles)	References
**Social Media (6)**	Network Data (2)	[355,356]
	Gender, Relationship Status, Sexual Orientation (1)	[354]
	Facebook Likes (2)	[10,11]
	Check-ins (1)	[170]
**Dating Website (1)**	Images (1)	[357]

The notion that individuals may unintentionally ‘leak’ clues to their sexuality in their digital traces may therefore be worrying to those who may want to keep such data private or hidden. In fact, all of the studies within our set examined inference from data that was unintentionally revealed by the individuals themselves or inferred through homophily [10,11,201,354–356]. For instance, Kosinski et al. [10] found that Facebook likes such as ‘Ellen DeGeneres’, ‘Mac Makeup’ and ‘Wicked The Musical’ were highly predictive of homosexual males, and ‘Not Being Pregnant’ and ‘No H8 Campaign’ were predictive of homosexual females. Further, ‘Being Confused After Waking Up From Naps’ and ‘Nike Basketball’ were highly predictive of heterosexual males, and ‘Adidas Originals’ and ‘Yahoo’ were predictive of heterosexual females.

Alternatively, research by Jernigan et al. [355], Sarigol et al. [356] and Garcia [354] used data derived from other people to infer individuals’ sexuality—their findings highlighted accuracies of around 00.80 (AUC). In particular, Sarigol et al. [356] and Garcia [354] demonstrated how such techniques could be used to infer the sexuality of non-users, also referred to as the ‘*shadow profile hypothesis’*. By analysing data from profiles on the (discontinued) social networking site Friendster, Sarigol et al. [356] and Garcia [354] found that sexual orientation groups were affected by network size and disclosure parameters where, as size/disclosure increases, so does the likelihood of inferring a non-user’s private data. Although there is limited work exploring shadow profiles, these findings highlight a concerning possibility that future research may want to consider when studying networks and individuals’ privacy. That is, whether it is possible to infer sexuality (or indeed any other attributes) from other peoples’ data, and in turn what can be done in order to protect peoples’ privacy.

### Other demographic attributes

Numerous articles reported multiple demographics that were distinct from the main traits outlined thus far. In most cases, these attributes were not studied independently and, (to our knowledge) do not have extensive research histories or theoretical backgrounds from social psychology. Nevertheless, we believe inferring these attributes forms an important part in profiling individuals, and are likely to receive more research attention in the future. Because of the limited literature surrounding the remaining attributes, we display the main findings for each in the series of tables that follow and in the supplementary materials. The attributes identified include: family and relationships (Table 11, Table 12, S7 Table), ethnicity and race (Table 13, Table 14, S8 Table), education (Table 15, Table 16, S9 Table), income (Table 17, Tables 18 and S10 Income), language (Table 19, Table 20, S11 Table), religion (Table 21, Table 22, S12 Table), occupation (Table 23, Table 24, S13 Table), health (Table 25, Tables 26 and S14) and social class (Table 27, Table 28, S15 Table).

**Table 11:** Number of articles predicting family and relationship status, with associated platforms and references.

Category (n = no. articles)	Platform (n = no. articles)	References
**Social Media (8)**	Facebook (3)	[10,11,156]
	Friendster (1)	[354]
	Twitter (4)	[20,84,87,358]
	Sina Weibo (1)	[170]
**Smartphone (9)**	NR (9)	[13,14,177,178,182,184,189,329,359]
**Websites (1)**	NR (1)	[200]

**Table 12:** Number of articles predicting family and relationship status, with associated predictors and references.

Category (n = no. articles)	Predictors (n = no. articles)	References
**Social Media (8)**	Facebook Likes (2)	[10,156]
	Language (4)	[20,84,87,358]
	Relationship Status (1)	[354]
	Network Data (1)	[358]
	Check-ins (1)	[170]
**Smartphone (9)**	Application Data, Behavioural Data, Call Data (8)	[13,14,177,178,182,189,329,359]
	Location Data (1)	[184]
**Websites (1)**	Web Usage Data (1)	[200]

**Table 13:** Number of articles predicting ethnicity or race, with associated platforms and references.

Category (n = no. articles)	Platform (n = no. articles)	References
**Social Media (15)**	Twitter (12)	[17,20,360,361,29,62,253,274,288,299,301,302]
	Facebook (3)	[10,158,330]
**Websites (3)**	News (1)	[362]
	Other (2)	[62,294]
**Devices (2)**	Smartphone (1)	[13]
	Tablet (2)	[193]
**Radio (1)**	Meta-data, Listening Habits (1)	[243]

**Table 14:** Number of articles predicting ethnicity or race, with associated predictors and references.

Category (n = no. articles)	Predictors (n = no. articles)	References
**Social Media (15)**	Names (6)	[29,51,158,301,330,360]
	Language (11)	[17,20,361,51,62,158,253,274,299,302,360]
	Network Data (2)	[51,62]
	Location Data (2)	[29,360]
	Meta-data (1)	[360]
	Facebook Likes (1)	[10]
	Profile Images (2)	[17,51]
**Websites (3)**	Names (1)	[362]
	Web Browsing Histories (1)	[294]
	Language, Network Data (1)	[62]
**Devices (2)**	Application Data (1)	[13]
	Actions, Keystrokes, Timestamps (1)	[193]
**Radio (1)**	Meta-data, Listening Habits (1)	[243]

**Table 15:** Number of articles predicting education level, with associated platforms and references.

Category (n = no. articles)	Platform (n = no. articles)	References
**Social Media (8)**	Twitter (6)	[20,62,84,253,254,358]
	Facebook (1)	[363]
	Sina Weibo (1)	[170]
**Websites (4)**	NR (4)	[62,197,200,294]
**Email (4)**	NR (4)	[238–241]
**Wi-Fi (1)**	NA (1)	[250]

**Table 16:** Number of articles predicting education level, with associated predictors and references.

Category (n = no. articles)	Predictors (n = no. articles)	References
**Social Media (8)**	Language (7)	[20,62,84,253,254,358,363]
	Network Data (2)	[62,358]
	Meta-data (2)	[358,363]
	Facebook Likes (1)	[363]
	Check-ins (1)	[170]
**Websites (4)**	Language (1)	[62]
	Network Data (1)	[62]
	Website Data (1)	[197]
	Meta-data (1)	[197]
	Web Browsing Histories (1)	[294]
	NR (1)	[200]
**Email (4)**	Language (4)	[238–241]
**Wi-Fi (1)**	Wi-Fi Traffic (1)	[250]

**Table 17:** Number of articles predicting income, with associated platforms and references.

Category (n = no. articles)	Platform (n = no. articles)	References
**Social Media (6)**	Twitter (6)	[20,84,253,285,302,364]
**Smartphone (5)**	NR (5)	[13,187,365–367]
**Websites (2)**	NR (2)	[200,294]

**Table 18:** Number of articles predicting income, with associated predictors and references.

Category (n = no. articles)	Predictors (n = no. articles)	References
**Social Media (6)**	Language (6)	[20,84,253,285,302,364]
**Smartphone (5)**	Application Data (1)	[13]
	Call/SMS Data (4)	[187,365–367]
	Network Data (1)	[187]
**Websites (2)**	Web Usage Data (2)	[200,294]

**Table 19:** Number of articles predicting language, with associated platforms and references.

Category (n = no. articles)	Platform (n = no. articles)	References
**Devices (2)**	Smartphone (1)	[329]
	Tablet (1)	[193]
**Blogs (1)**	Blogger.com (1)	[295]
**Email (3)**	NR (3)	[237–239]
**Social Media (3)**	Twitter (3)	[29,89,92]

**Table 20:** Number of articles predicting language, with associated predictors and references.

Category (n = no. articles)	Platform (n = no. articles)	References
**Devices (2)**	Application Data (1)	[329]
	Actions, Keystrokes, Timestamps (1)	[193]
**Blogs (1)**	Language (1)	[295]
	Language (3)	[237–239]
	Names, Location (1)	[29]
	Language (2)	[89,92]

**Table 21:** Number of articles predicting religion, with associated platforms and references.

Category (n = no. articles)	Platform (n = no. articles)	References
**Social Media (6)**	Twitter (4)	[20,67,84,368]
	Facebook (2)	[10,11]
**Search Engines (1)**	Bing (1)	[9]
**Smartphones (1)**	NR (1)	[329]

**Table 22:** Number of articles predicting religion, with associated predictors and references.

Category (n = no. articles)	Platform (n = no. articles)	References
**Social Media (6)**	Language (4)	[20,67,84,368]
	Facebook Likes (2)	[10,11]
**Search Engines (1)**	Facebook Likes, Profile Data (1)	[9]
**Smartphones (1)**	Application Data (1)	[329]

**Table 23:** Number of articles predicting occupation, with associated platforms and references.

Category (n = no. articles)	Platform (n = no. articles)	References
**Social Media (10)**	Twitter (10)	[66,73,87,89,91,92,209,358,369,370]
**Blogs (2)**	NR (2)	[209,233]
**Smartphones (8)**	NR (8)	[14,177,178,182–184,188,189]
**Websites (1)**	NR (1)	[197]
**Forums (1)**	NR (1)	[276]

**Table 24:** Number of articles predicting occupation, with associated predictors and references.

Category (n = no. articles)	Platform (n = no. articles)	References
**Social Media (10)**	Language (9)	[66,73,87,91,92,209,358,369,370]
	Network Data (3)	[66,358,370]
	Meta-data (5)	[66,209,303,358,370]
**Blogs (2)**	Language (2)	[209,233]
	Meta-data (1)	[209]
**Smartphones (8)**	Application Data (6)	[14,177,178,182,188,189]
	Call Data (5)	[14,178,182,188,189]
	Location Data (2)	[183,184]
**Websites (1)**	Time/Day Data, Website Data (1)	[197]
**Forums (1)**	Language (1)	[276]

**Table 25:** Number of articles predicting health, with associated predictors and references.

Category (n = no. articles)	Platform (n = no. articles)	References
**Social Media (7)**	Twitter (5)	[20,67,84,371,372]
	Facebook (1)	[10]
	MyFitnessPal (1)	[373]
	Reddit (1)	[374]
**Smartphones (2)**	NR (2)	[176,201]

**Table 26:** Number of articles predicting health, with associated predictors and references.

Category (n = no. articles)	Predictors (n = no. articles)	References
**Social Media (7)**	Language (5)	[20,67,84,371,372]
	Images (1)	[374]
	Facebook Likes (1)	[10]
	Behavioural Data (1)	[176]
**Smartphones (2)**	Application Data (2)	[176,201]
	Network Data (1)	[201]

**Table 27:** Number of articles predicting social class, with associated platforms and references.

Category (n = no. articles)	Platform (n = no. articles)	References
**Social Media (1)**	Twitter (1)	[375]
	Foursquare (1)	[375]

**Table 28:** Number of articles predicting social class, with associated predictors and references.

Category (n = no. articles)	Platform (n = no. articles)	References
**Social Media (1)**	Language (1) [375]	[375]

## Discussion

The ability to predict individuals’ demographic attributes from their online activity has many useful applications including marketing, criminal investigations, monitoring societal events and tracking health. Academic research attempting to use computational methods to infer attributes has proliferated in recent years and overall has demonstrated reasonable degrees of accuracy. This systematic review has highlighted the current state-osf-the art with regards to demographic prediction, in terms of the platforms, digital traces and methods currently employed. To date, age and gender are the most studied demographics—perhaps this is due to more established research histories within the social psychology literature, compared to other attributes.

A key factor in predicting such information is the type of digital footprint from which this information is derived. Many studies that perform linguistic analyses highlight trends in patterns of language use (in terms of style, content, slang etc.) that seem common across platforms and traits. For instance, females tend to use words such as *shopping*, *excited*, *sooo*, *yay <3*, e.g. [20,160,207], and males tend to use words such as *I’ve*, *fuck*, *league*, *youtube*.*com*, *system*, *software*, e.g. [18,20,160]. Younger adults tend to use shorter sentences and words such as *cuz*, *haha*, *school*, *don’t*, *office*, *beer*, e.g. [20,160], and older adults (typically classified as over 30) tend to use words such as *kids*, *family*, *daughter*, *don’t*, e.g. [160,207]. However, rarely are differences in either age or gender connected to theoretical perspectives on either life span development, or gender. For instance, there is considerable previous (earlier) work on the use of hedges and tag questions (e.g. it’s a nice day, *isn’t it*?) by female speakers, and how such language may reflect power differentials and inequalities in a patriarchal society, e.g. [265].

Similarly, differences in the challenges faced across life stages have been widely theorised, e.g. [376], as have the changing goals that people strive for as they age, e.g. [377,378]. However, it was rare to find consideration of *what* the predictive features might mean to a social scientist within the papers reviewed, and often the predictive features were not even mentioned in the paper, making connection to social theory impossible. Instead, much of the time the approach taken was to compare classifiers, and to allow the machine learning program to identify the best features (or to include as many as possible in a training set, and then replicate with the ‘best’ features in a kept back sample for validation purposes. Although in many cases this likely results from conventions in different research fields–computer science approaches tend to focus more on successful methods and prediction, whereas psychology emphasises causes and explanations (for a detailed discussion of this, see the work by Yarkoni and Westfall [379]).

Network data, in the form of metrics derived from social network neighbours, structural features and popularity (e.g. mentions, follows) were also useful for predicting a range of attributes including age, gender, location and sexual orientation, e.g. [252,312,315,355]. The ability to use network data to infer attributes can be incredibly useful in identifying information that may not be disclosed directly by an individual. However, this has serious implications for privacy–individuals may want to keep their political beliefs, sexuality etc. private and may not realise they are inadvertently revealing them through their digital activity. Alternatively, the extent to which this is a concern is dependent on who the individual would want to conceal such information from–computer algorithms may be able to detect such information; however, it is unlikely that the average human or people within their network would be able to make such inferences accurately from looking at this type of data.

One aspect that was noticeable from the studies presented is that there was no focus on the more complex modes of interaction, such as deception or attempts by individuals to present themselves differently at different points in time/in different contexts. For instance, an individual’s language is likely to differ when talking to friends in comparison to writing an online review. Would a computer be able to identify their demographic attributes as being the same across both contexts? Research on communication accommodation demonstrates that individuals co-ordinate their language use with those they are conversing with, e.g. [279,380], suggesting that the assessment of demographics from, say, the language used, should be more difficult in the context of interactions if one person’s use of specific language is influenced by their conversational partners’ use of the same linguistic features. Moreover, the degree to which people accommodate towards their conversational partner is influenced by a number of factors, including power differentials [381]. Indeed, there is evidence that deception in text-based communication can be identified by the language used by the person *being lied to* as well as via changes in the language of the deceiver [382] suggesting that analysing language from interactions as individual data points needs to be treated with particular caution. Future work could attempt to decipher whether computer models are able to use similar sociolinguistic techniques to infer attributes from these types of interactions, and to unpick individual level characteristics from those dependent on the nature of the interaction or audience.

We also suspect that rather than simply comparing the effectiveness of classification algorithms, or mechanical turk workers vs. a classifier, in the future authors may wish to take a more theoretically driven approach to feature selection. For instance, there is considerable evidence that pronoun use can be linked to a number of social and psychological theories–including ingroup (‘we’) and outgroup (‘they’) identification (e.g.[383]), leadership ([384]) and gender bias [385]. Given the existing body of work identifying differences between groups based on these features, one would expect that a classifier *should* be able to distinguish between categories based on existing theory. It would also further our understanding of an existing body of work if a theoretically derived model were compared against a ‘best feature’ model derived from a machine learning approach.

Finally, in reviewing the papers herein it became clear that summarising the results of studies across labs is particularly difficult. In many cases multiple, different algorithms are used, the most discriminating features aren’t reported, or simple accuracy statistics are reported without the full confusion matrix or recall / sensitivity information provided. We would strongly advise that the field consider methods to standardise reporting across studies and labs, enabling replication and for future studies to build more ably from the basis of earlier work.

## Supporting information

S1 Table PRISMA checklist for systematic review. (DOCX)

S2 Table Gender articles and data. Note: All accuracy measures in this database (and those below) are summarised in ranges (lowest to highest) and are reported to 2 decimal places. This was performed in order to standardise the varied styles of reporting provided in the set of articles. Further, some articles reported their findings as graphs or other visualisations, meaning that we could not extract specific accuracies. In these instances, cells are left blank. For instances where data were simply not reported in an article, we denote this with NR (i.e. Not Reported).
(XLSX)

S3 Table Age articles and data. (XLSX)

S4 Table Location articles and data. (XLSX)

S5 Table Political orientation articles and data. (XLSX)

S6 Table Sexual orientation articles and data. (XLSX)

S7 Table Family and relationships articles and data. (XLSX)

S8 Table Ethnicity and race articles and data. (XLSX)

S9 Table Education articles and data. (XLSX)

S10 Table Income articles and data. (XLSX)

S11 Table Language articles and data. (XLSX)

S12 Table Religion articles and data. (XLSX)

S13 Table Occupation articles and data. (XLSX)

S14 Table Health articles and data. (XLSX)

S15 Table Social class articles and data. (XLSX)

S16 Table Classifier codes. (XLSX)
